# The Autoimmune Tautology: An In Silico Approach

**DOI:** 10.1155/2012/792106

**Published:** 2012-03-05

**Authors:** Ricardo A. Cifuentes, Daniel Restrepo-Montoya, Juan-Manuel Anaya

**Affiliations:** ^1^Center for Autoimmune Diseases Research (CREA), School of Medicine and Health Sciences, Universidad del Rosario, Carrera 24, No. 63-69 piso 3, Bogotá, Colombia; ^2^Bioinformatics and Intelligent Systems Research Laboratory (BIOLISI), Universidad Nacional, Avenida Carrera 30, No. 45-03, Bogotá, Colombia

## Abstract

There is genetic evidence of similarities and differences among autoimmune diseases (AIDs) that warrants looking at a general panorama of what has been published. Thus, our aim was to determine the main shared genes and to what extent they contribute to building clusters of AIDs. We combined a text-mining approach to build clusters of genetic concept profiles (GCPs) from the literature in MedLine with knowledge of protein-protein interactions to confirm if genes in GCP encode proteins that truly interact. We found three clusters in which the genes with the highest contribution encoded proteins that showed strong and specific interactions. After projecting the AIDs on a plane, two clusters could be discerned: Sjögren's syndrome—systemic lupus erythematosus, and autoimmune thyroid disease—type1 diabetes—rheumatoid arthritis. Our results support the common origin of AIDs and the role of genes involved in apoptosis such as *CTLA4*, *FASLG,* and *IL10*.

## 1. Introduction

There are clinical and genetic grounds for assuming similar immunogenetic mechanisms in autoimmune diseases (AIDs). Clinical evidence highlights the cooccurrence of distinct AIDs within members of a nuclear family and within an individual [1]. Individuals with a multiple autoimmune syndrome (MAS) have been grouped into three basic groups in which various AIDs cluster around one of three “main” AIDs, namely, systemic lupus erythematosus (SLE), autoimmune thyroid disease (AITD), and primary Sjögren's syndrome (SS). These three might be considered the “chaperones” of the other AID [2]. Along the same line of clinical evidence, there are therapies such as tumor necrosis factor inhibitors, rituximab, or a gluten-free diet that are already proving effective for more than one AID [3, 4]. With regards to genetic evidence, it has also been stated that around 44% of the single nucleotide polymorphisms (SNPs), which were found in genome-wide association studies (GWAS) on AIDs, are shared by two or more of the following diseases: celiac disease, Crohn's disease, psoriasis, multiple sclerosis (MS), rheumatoid arthritis (RA), type 1 diabetes (T1D), and SLE [5].

There are also genetic differences among AIDs. In spite of sharing several susceptibility genes, the differences among most AIDs, in particular systemic ones such as SLE and RA, seem to reside in the contribution of each gene to each disease [6]. Additionally, clusters of AIDs have been described where SNPs that make an individual susceptible to one class of AIDs also protect from another class of AIDs [7]. Furthermore, it is already known that different AIDs are associated with some different alleles from the human leukocyte antigen (HLA) [6].

As a consequence, it is important to obtain a general panorama of the problem in order to understand the origin of the AIDs. However, in biomedical research, the amount of experimental data and published scientific information is overwhelming. Therefore, literature-based discovery (LBD) tools emerge as useful to make the biomedical literature accessible for research purposes [8]. Thus, different LBD methods have been used to mine large amounts of literature and find the necessary information (Table 1) [8–11] with two main approaches in the biomedical domain [12]. One approach focuses on the extraction of precise relationships between concepts, and the other relates biomedical concepts one to each other based on the statistical properties of their occurrence and cooccurrence in literature. A known LBD method based on concept occurrence is the concept profile (CP), in which a concept is characterized by a list of associated concepts, together with weights that indicate the strength of the association [13]. 

The output of the concept profiling method is a list of associations ordered by the strength of their relationship that needs verification. It is typically done with domain-relevant knowledge usually based on expert human judgments or even experimental validation [8, 14]. The latter approach is currently more feasible in the biomedical field given the increase in experimentally identified binary interactions between proteins that has made it possible to see how these components come together to form large functional regulatory networks [15]. There are several network approaches [16] that could be organized based on the type of biological or molecular interactions [17] and that analyze diverse databases (Table 2) [18–24]. Thus, the information related to protein-protein interactions helps us to study these associations from the perspective of biochemistry, signal transduction, and biomolecular networks [25]. Identification of functional roles of unknown pathogenic genes can also make it possible to understand pathogenic mechanisms. Proteins that are tightly connected in biological networks often work in similar processes [26].

This complex panorama shows that we are still distant from knowing everything, that is to know about genes, their interactions with other genes, and their impact on biological functions [6]. Therefore, the aim of this study was to obtain information from the literature and annotated databases to find main common genes in autoimmunity and determine to what extent they contribute to different clusters of AIDs.

## 2. Methods

**Figure 1:**
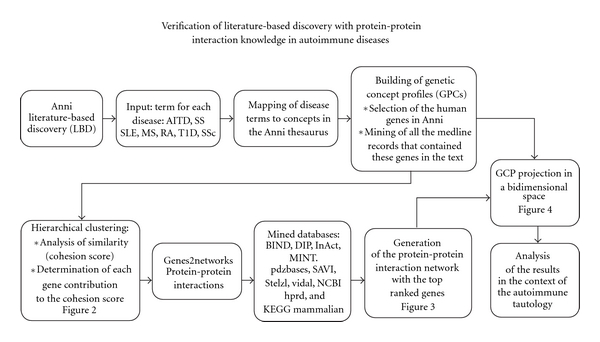
Flowchart of the methodology. AITD: autoimmune thyroid disease, SS: primary Sjögren's syndrome, SLE: namely systemic lupus erythematosus, MS: multiple sclerosis, RA: rheumatoid arthritis, T1D: type 1 diabetes, and SSc: systemic sclerosis.

Our analysis was made by using experimental knowledge of protein-protein interaction to evaluate the top ranked genes, which had been found through the CP approach to mine the biomedical literature (Figure 1).

### 2.1. Literature-Based Knowledge Discovery

The concepts selected as input for the LBD software were the three AIDs referred to as chaperones of autoimmunity (i.e., AITD, SS, and SLE). We also selected as input concepts the AIDs mentioned in literature as present in relatives of probands of these three diseases: MS, RA, T1D, vitiligo (VIT), and systemic sclerosis (SSc) [2].

To evaluate the genetic similarity of those AIDs, we chose the Anni software because it uses the CP methodology that has proven to be effective for finding information in the form of associations in the biological domain [27]. First, the mapping of those concepts in the thesaurus of the Anni software that uses the concept profile methodology was evaluated [28]. At this point, we eliminated the VIT concept because it showed ambiguity in mapping. Next, the CP for each one of the seven remaining AIDs was built. Those profiles corresponded to the weighted list made by all the genes mentioned in MedLine, so they were called genetic CPs (GCPs). To do this, we selected the 25.010 genes that belong to human beings from the thesaurus in Anni, and, then, we mined all the MedLine records that contained these genes in their text. Next, the associations between GCP were explored through hierarchical clustering. The clusters were generated by matching the GCP for each one of the mapped AIDs, as the CP can be described as vectors. Then, the similarities between the GCP in the found clusters were analyzed. For this purpose, we obtained a cohesion score by using as an inclusive filter for matching the described 25.010 genes. Briefly, the cohesion score is an average of the inner products of all possible pairs of profiles corresponding to the concepts in the group of interest. The contribution of each gene in the profile to the cohesion score was assessed in terms of percentage. To interpret the cohesion score we used a *P* value that gives the probability that the same score or higher would be found in a random group of the same size. This *P*-value was obtained by using the default parameter in Anni of 200 iterations. Finally, the distances between concepts that reflect the matching value between GCPs were projected in a two-dimensional space, in order to understand the AID clustering.

### 2.2. Network Analysis

To analyze if the genes in the clusters previously found through LBD corresponded to proteins with a known interaction, a network analysis was done with the genes that contributed at least 0.1% to any of the clusters found by the method described in Section 2.1. For this purpose, the software, Genes2networks, was selected because it finds relationships between proteins by using ten high quality mammalian protein-protein interaction databases that take into account not only filtered high throughput but also low throughput experiments that have a lower probability of false positives [29]. Then, in order to find tightly connected proteins, the settings that were used in Genes2networks to build the network were (1) no filter for minimum number of references, (2) the maximum links per reference were four, (3) a maximum pathway length of two, and (4) a significant Zscore of 2.5 of the intermediate nodes, which was calculated through a binomial proportions test, as previously described [29].

### 2.3. Systematic Search

We did a classical systematic search, as previously done by our group [30], to understand the relevance of the genes found by our approach on AIDs. The genes selected were ones that contributed more than 1% to two or more clusters of AIDs and were close to each other in subnetworks where they were separated by a maximum of one node. To do this, we did a systematic search of the Catalog of Published Genome-Wide Association Studies at http://www.genome.gov/26525384 and on PubMed by using three terms: the gene name, the MeSH term “genome-wide association study" and the MeSH term for each AIDs that belonged to the found clusters. Consequently, the terms for the AIDs were chosen from the next MeSH terms: “arthritis, rheumatoid," “multiple sclerosis,” “diabetes mellitus, type 1," “lupus erythematosus, systemic," “scleroderma, systemic” and “Sjögren's syndrome." In the case of thyroid disease, the term “thyroid” was used. The information from PubMed was excluded when the retrieved information did not explicitly refer to the specific gene, for instance when CD4 referred to a type of cell (i.e., lymphocyte) but not to the gene.

## 3. Results

**Figure 2:**
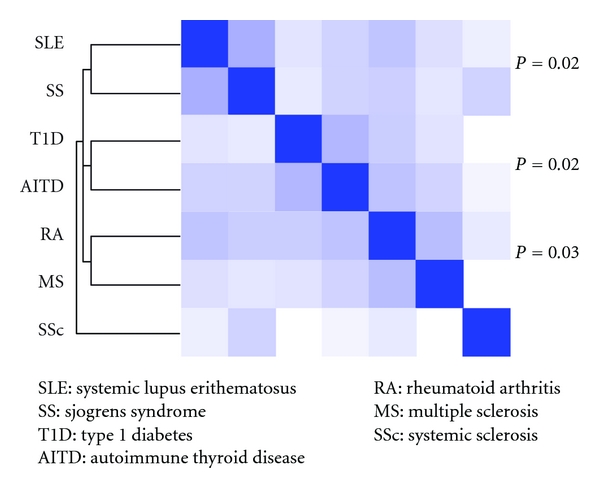
Clustering of seven autoimmune diseases. SLE: systemic lupus erithematosus, SS: Sjögren's syndrome, T1D: type 1 diabetes, AITD: autoimmune thyroid disease, RA: rheumatoid arthritis, MS: multiple sclerosis, SSc: systemic sclerosis.

There were three paired clusters with a probability equal to or less than 3 percent that their cohesion score would be found in a random group of the same size: SLE with SS (*P* = 0.02), T1D with AITD (*P* = 0.02), and RA with MS (*P* = 0.03) (Figure 2). Regarding the genes that contributed to building the clusters, 55 of them had a contribution higher than 0.1% to the cohesion score of any of those clusters. Some of them were shared by more than one cluster:* HLA-DQB1, CD4, TNFSF25, FASLG, IL1B, IL6, IL10, TNFSF13B, CTLA4 and HLA-DRB1*. The later three had a contribution higher than 20% to any of the three specific clusters. The other genes contributed to only one cluster. It should be mentioned that there were also specific genes for one cluster that had a contribution of around 20% to their clusters, such as *TRIM21* and *TROVE2* in the cluster made up of SLE and SS, *TPO* in the cluster made up of T1D and AITD, and *TNF* in the cluster made up of RA and MS (Table 3).

**Figure 3:**
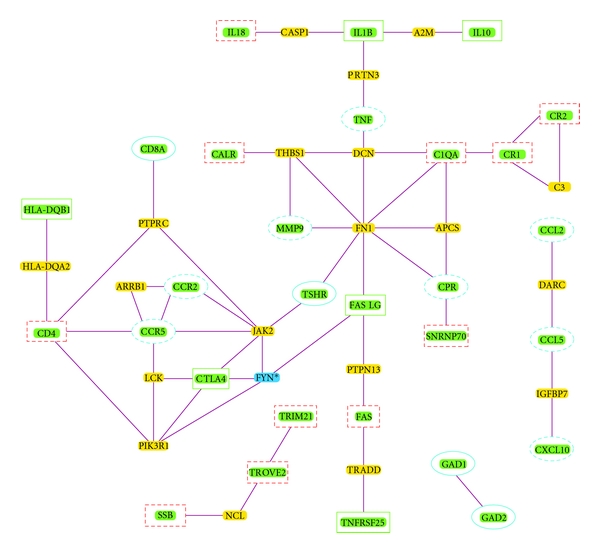
Network analysis of the genes that contribute to the clusters of autoimmune diseases. Solid squares: genes with a contribution higher than 0.1% that are shared by more than one cluster. Dotted squares: genes with a contribution higher than 0.1% from the SLE-SS cluster. Solid ovales: genes with a contribution higher than 0.1% from the T1D-AITD cluster. Dotted ovales: genes with a contribution higher than 0.1% from the RA-MS cluster. The other nodes correspond to significant intermediary ones (the asterisk indicates a nonsignificant intermediary node).

Concerning to the network analysis, we used as input the previously mentioned 55 genes. 29 of these 55 entries were identified and described on the graph (Figure 3). Some genes such as *IL6* and *HLA-DRB1* did not appear in the network. This could have been because of the strict threshold, a maximum pathway length of two, established to avoid weak interactions or because they did not have protein-protein interactions already reported in the used database. For instance, some genes relating to antigen presentation such as *HLA-DRB1 *may be absent in protein interaction networks.

The network had 20 intermediary nodes, 19 significant with a Z score above the cutoff of 2.5 (Table 4), thus indicating that they may be specific to interact with components from the inputted seed list of genes. In other words, those results indicated that the seed genes encode proteins that had strong and specific interactions. In the graph, it can also be seen that the genes common to more than one cluster belonged to the same connected network (Figure 3). There were two subnetworks of genes that had a contribution higher than 0.1% and that were shared by more than one cluster. The first was made up of* HLA-DQB1*, *CD4*, *CTLA4* and *FASLG* that were genes connected through only one internode (*TNFRSF25* is also connected through three internodes with *FASLG*) and the second subnetwork was made up of *IL1B* and *IL10* that was connected to TNF, the gene with the highest contribution to the cluster made by RA and MS. There was also another subnetwork made with the directly connected *C1QA, CR1, and CR2* genes that belonged to the cluster made by SLE and SS (Figure 3).

We also observed that some of the genes with a contribution higher than 0.1% to only one cluster belonged to three little separate networks. The first little network had the genes *GAD1* and *GAD2* from the cluster of T1D-AITD, the second had the sgenes *TRIM21*, *TROVE2,* and *SSB* from the cluster of SLE-SS, and the third had the genes *CCL5* and *CCL2* from the cluster RA-MS (Figure 3).

Through the systematic search, we looked for GWAS information on six genes (Table 5). *HLA-DQB1* [31]*, CTLA4 *[32, 33], and* FASLG *and *IL10* [34] were related to AIDs in GWAS. In contrast, to date *CD4 and IL1B* have not been related by GWAS data to any of the above-mentioned AIDs.

**Figure 4:**
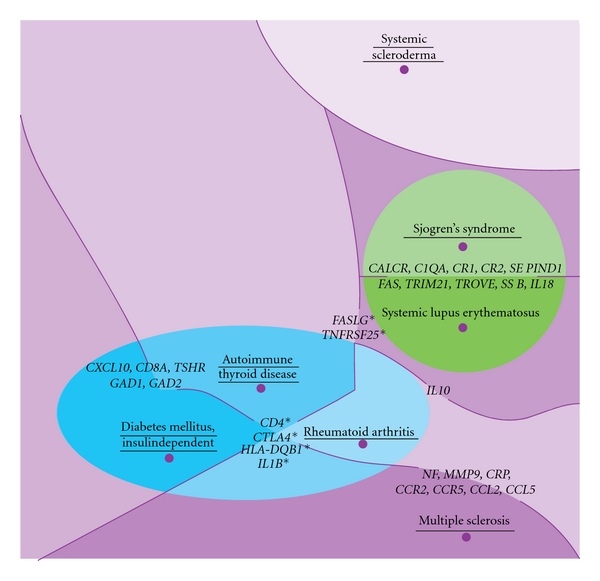
Projection of the seven studied autoimmune diseases on a plane. This figure shows the shared space of the genetic concept profiles from the studied AIDs (underlined), according to the matching value of their genetic concept profiles. We can see the genes with a contribution to clustering higher than 0.2%, the asterisk indicates the genes shared by two clusters.

Finally, according to the distances obtained through the LBD approach, the evaluated AIDs were projected into two main spaces that are near each other. The first included SS and SLE, and the second, AITD, T1D, and RA. Both were distant from SSc and a little closer to MS, especially in the case of the RA (Figure 4).

## 4. Discussion

Our *in silico* approach that combined LBD and network analysis of protein-protein interactions allowed us to confirm common genes involved in autoimmunity as well as to estimate their contribution into the clusters of AIDs. Some common genes made an important contribution to only one specific cluster such as *TRIM21*, *TROVE2,* or *SSB*, but others were present in more clusters of AIDs such as *HLA-DQB1*, *FASLG*, *CTLA4,* or *CD4*. However, our approach did not intend to find all the genes shared among AIDs. In fact, not all the genes could be validated through protein-protein interactions, and others did not make a significant contribution to the described clusters of AIDs.

With regards to genes shared by more than one cluster of AIDs, it can be seen that they were typically found to be significant in GWAS. However, there were exceptions. In the case of *CD4*, an association was not found with any AID by GWAS, but another approach that combines biological similarities found that *CD4* is a likely causal gene of RA [35], one that had been seen as high risk by recent studies [36, 37]. In contrast to GWAS, the genes that were found to be related to RA by the approach that combines biological similarities could be easily classified into related functional categories or biological processes [35], thus making these finding similar to our results.

In contrast, there were genes that contributed mainly to specific clusters of AIDs such as *TRIM21* (*Ro52*), *TROVE2* (*Ro60*) and *SSB* (*La*) that were found to be important for the SLE-SS cluster. In spite of the fact that they were not significant at the GWAS level, this observation agreed with the fact that anti-SS-A (Ro52/Ro60) autoantibodies have been described as serological markers for both SS and SLE [38–40]. Ro52 works as an E3 ligase and mediates ubiquitination of several members of the interferon regulatory factor (IRF) family. Its deficiency has been associated with enhanced production of proinflammatory cytokines that are regulated by the IRF transcription factors including cytokines involved in the Th17 pathway [41]. Although Ro ribonucleoproteins such as Ro60 and La were discovered many years ago, their function is still poorly understood [42]. It has been suggested that *TROVE2* acts as a modulator in the Y RNA-derived miRNA biogenesis pathway. The hypothesis is that Ro RNPs are “latent” pre-miRNAs that can be converted into miRNAs under certain circumstances [42]. In addition, it was observed that narrow-band ultraviolet B irradiation provoked significant alterations of the keratinocyte morphology and led to the membrane expression of antigens recognized by anti-La and anti-Ro 60 kDa sera [43].

Another observation about genes that contributed mainly to specific clusters was that genes typically involved in one AID such as C1QA and CR1 in the case of SLE, or GAD1 and GAD 2 in the case of T1D, were found by our approach to be shared with SS or AITD, respectively. These findings agree with the observations that around 24% of patients with T1D expressed antithyroid autoantibodies and that 17% of them had AITD in comparison to 6% of age-matched controls [44].

The projection of the AIDs on a plane agreed with the similarity between genetic variation profiles of T1D and AITD found by another approach, which builds genetic variation profiles taking into account *P* values and odds-ratios of significant SNPs in GWAS, but does not totally agree with the claimed opposition between MS and RA [7]. It can be seen that RA has some similarity with MS in spite of being closer to AITD. This projection also agreed with the behavior of HLA, even in admixed Latin-American populations, as diseases that were closer in it shared risk alleles. This is the case for SLE, SS, and T1D that have the DRB1*03:01 allele as a risk factor [30, 45, 46]. Furthermore, in diseases that are distant in our clustering analysis, such as MS and T1D, the same DQB1*06:02 allele gives protection to the first but risk to the second disease [47].

From the biological perspective, our results showed the central role of *FASLG* as it is connected through one node to *CTLA4*, which is connected to *CD4* through one node and that, in turn, is connected to *HLA-DQB1* the same way (Figure 3). *FASLG* is also connected with *TNF* through two nodes, and this is connected, in turn, through one node to *IL1B*, which is also connected through one node to *IL10* and *IL18*. It is interesting that these two pathways are involved in similar processes since *CTLA4,* and *IL10* are implicated in peripheral immunologic tolerance [48]. *FASLG* is also connected to two other pathways. It is connected through one node to *C1QA, *which is directly connected to *CR1. *Lastly, it is also indirectly connected to the pathway of *TROVE2, TRIM21,* and* SSB* through a route that was not shown on the graph. This route involved *SUMO1*, a gene that has been associated with a blockage of the FAS pathway in RA, thus preventing apoptosis [49]. Taken together, our results highlight the autoimmunity role of genes involved in the process of apoptosis such as *CTLA4*,* FASLG, *and *IL10* that work together with genes involved in the inflammatory process such as *IL1B* [50].

Biomedical informatics involves a core set of methodologies that can provide a foundation for crossing the “translational barriers" associated with translational medicine [51]. Since the classical systematic review of literature could be incomplete because a significant amount of conceptual information present in literature is missing from the manually indexed terms [10], it seems to be advisable to combine the classical approach for searching literature with these new techniques.

In summary, the bioinformatics approach that combines text mining and network analysis of proteins allowed functional modules of interacting disease genes to be identified and can be used to predict additional disease gene candidates. Our approach also gave further evidence of the common origin of AIDs as the clustering of these diseases took into account thousands of genes that contribute to make the genetic concept profiles. Furthermore, this mining approach identified the specific contribution of a number of genes to causing some AIDs to cluster. These genes could be useful for further research.

## Figures and Tables

**Table 1:** Examples of literature-based knowledge discovery tools.

Tool	Mined data	URL
ANNI	MedLine	http://www.biosemantics.org
Arrowsmith^1^,	MedLine, OVID	http://wiki.uchicago.edu/
	UMLS concepts in	
Arrowsmith^2^	title words (MedLine)	http://arrowsmith.psych.uic.edu/
BITOLA	MeSH and LocusLink	http://www.mf.uni-lj.si/bitola/
LitLinker	UMLS	http://litlinker.ischool.washington.edu/
FACTA	MedLine	http://refine1-nactem.mc.man.ac.uk/facta/
FAUN	MedLine	https://grits.eecs.utk.edu/faun/

1 University of Chicago

2 University of Illinois at Chicago

For more information about biomedical text mining tools visit

http://arrowsmith.psych.uic.edu/arrowsmith_uic/tools.html.

**Table 2:** Examples of tools to analyze biological pathways.

Tool	Analyzed data	URL
Cytoscape	220 diverse databases.	http://www.cytoscape.org/
BIANA	uniprot, GenBank, IntAct,	http://sbi.imim.es/web/BIANA.php
	KEGG and PFAM.	
Pathway studio	MedLine.	http://www.ariadnegenomics.com/products/pathway-studio/expression-analysis/algorithms
Patika	Reactome, UniProt, Entrez	http://www.patika.org/
	Gene, and GO.	
		
Genes2networks	BIND, DIP, IntAct, MINT,	http://actin.pharm.mssm.edu/genes2networks/
	pdzbase, SAVI, Stelzl, vidal, ncbi hprd, and KEGG mammalian	

**Table 3:** Genes with a contribution higher than 0.1% to the found clusters of the studied autoimmune diseases.

Cluster 1. SLE-SS		Cluster 2. T1D-AITD		Cluster 4. RA-MS	
Gene	%	Gene	%	Gene	%
*TRIM21*	27.91	*TPO*	32.4	*TNF*	39.5
*TNFSF13B*	27.46	*CTLA4*	28.6	*HLA-DRB1*	20.7
*TROVE2*	19.8	*TNFRSF25*	6.7	*IL10*	5.2
*SSB*	6.6	*HLA-DRB1*	6.7	*IL6*	2.2
*FAS*	2.7	*PTPN22*	6.4	*CCL2*	0.6
*DLAT*	2.6	*GAD1*	4.6	*CD4*	0.6
*IRF5*	1.0	*GAD2*	3.6	*MMP9*	0.6
*IL10*	0.9	*AIRE*	1.7	*IL1B*	0.5
*FASLG*	0.8	*PTPRN*	1.5	*IL4*	0.5
*TNFRSF25*	0.6	*HLA-DQB1*	0.5	*TNFSF13B*	0.5
*CR1*	0.5	*IDDM2*	0.5	*IL23A*	0.4
*CALR*	0.5	*SUMO4*	0.5	*CCR2*	0.4
*SPTAN1*	0.4	*ICA1*	0.4	*IL1RN*	0.4
*RNPC3*	0.4	*FOXP3*	0.3	*CCL5*	0.3
*CR2*	0.2	*FCRL3*	0.2	*ICAM1*	0.3
*SNRNP70*	0.2	*CD4*	0.2	*CXCR3*	0.3
*SERPIND1*	0.2	*FASLG*	0.2	*HLA-DQB1*	0.3
*C1QA*	0.2	*CXCL10*	0.2	*VCAM1*	0.2
*IL18*	0.2	*CD8A*	0.2	*CTLA4*	0.2
*IL6*	0.2	*IL1B*	0.2	*PADI4*	0.2
		*TSHR*	0.2	*IFNB1*	0.2
				*CRP*	0.2
				*CCR5*	0.2
				*IL12B*	0.2

SLE: systemic lupus erithematosus, SS: Sjögren's syndrome, T1D: type 1 diabetes, AITD: autoimmune thyroid disease, RA: rheumatoid arthritis, MS: multiple sclerosis, %: percentage of contribution to the cluster.

**Table 4:** Significance of intermediates sorted by z-score.

Gene name	Link	Link in background	Links to seed	Links in subnetwork	z-score
HLA-DQA2	3	11429	2	60	15,852
DARC	4	11429	2	60	13,692
LCK	67	11429	6	60	9,548
PRTN3	9	11429	2	60	9,007
APCS	10	11429	2	60	8,522
FN1	62	11429	5	60	8,215
IGFBP7	11	11429	2	60	8,103
PTPN13	12	11429	2	60	7,737
CASP1	18	11429	2	60	6,215
A2M	24	11429	2	60	5,293
DCN	25	11429	2	60	5,171
NCL	30	11429	2	60	4,655
C3	31	11429	2	60	4,566
JAK2	116	11429	4	60	4,356
PTPRC	35	11429	2	60	4,248
THBS1	37	11429	2	60	4,108
ARRB1	44	11429	2	60	3,690
TRADD	63	11429	2	60	2,910
PIK3R1	133	11429	3	60	2,761
FYN	153	11429	3	60	2,457

**Table 5:** Relevance on autoimmunity GWAS of the genes with a contribution higher than 1% to two or more clusters of the studied autoimmune diseases.

Gene	Full name	Location	GWAS catalogue	Reference
*HLA-DQB1*	Major histocompatibility complex, class II, DQ beta 1	6p21.3	MS, PBC, RA, SSc, CD, UC, CrD	[31]
*CD4*	CD4 molecule	12pter-p12	—	—
*CTLA4*	Cytotoxic T-lymphocyte-associated protein 4	2q33	T1D, RA, MS, SLE, CD	[32, 33]
*FASLG*	Fas ligand (TNF superfamily, member 6)	1q23	CD, CrD	—
*IL1B*	Interleukin 1, beta	2q14	—	—
*IL10*	Interleukin 10	1q31-q32	T1D, SLE, UC, CrD	[34]

MS: multiple sclerosis, PBC: primary biliar cirrhosis, RA: rheumatoid arthitis, SSc: systemic sclerosis, CD: celiac disease, CrD: crohn disease, T1D: Type 1 diabetes, SLE: systemic lupus erithematosus, UC: ulcerative colitis, PSO: Psoriasis.

## Abbreviations

AIDs: Autoimmune diseases
AITD: Autoimmune thyroid disease
CP: Concept profile
GCP: Genetic concept profiles
GWAS: Genome-wide association studies
HLA: Human leukocyte antigen
IRF: Interferon regulatory factor
LBD: Literature-based discovery
MAS: Multiple autoimmune syndrome
MS: Multiple sclerosis
RA: Rheumatoid arthritis
SLE: Systemic lupus erythematosus
SNPs: Single nucleotide polymorphisms
SS: Primary Sjögren's syndrome
SSc: Systemic sclerosis
T1D: Type 1 diabetes
VIT: Vitiligo.
